# Evaluation of *Kluyveromyces* spp. for conversion of lactose in different types of whey from dairy processing waste into ethanol

**DOI:** 10.3389/fmicb.2023.1208284

**Published:** 2023-08-08

**Authors:** Ashley Mae Ohstrom, Autumn Elizabeth Buck, Xue Du, Josephine Wee

**Affiliations:** Department of Food Science, The Pennsylvania State University, University Park, PA, United States

**Keywords:** whey, fermentation, lactose, *Kluyveromyces*, waste, ethanol

## Abstract

The processing of dairy products currently generates significant amounts of waste, particularly in the form of liquid whey. The disposal of whey poses a challenge to the environment due to its high organic content and biological oxygen demand. Whey contains lactose, soluble proteins, lipids, and minerals. While *Saccharomyces cerevisiae* can efficiently utilize glucose, they are unable to metabolize lactose. In contrast, *Kluyveromyces* spp. encode two genes, *Lac12* and *Lac4* that enable conversion of lactose to other by-products such as ethanol. Here, we selected five *Kluyveromyces* yeast inoculated into three different types of whey substrates, cheddar sweet whey, cream cheese acid whey, and yogurt acid whey that could be used to convert lactose into ethanol. We demonstrate that differences exist in ethanol production across different whey substrates inoculated with *Kluyveromyces* yeast. In sweet whey, *K. lactis, K. lactis* Y-1205 and *K. lactis* Y-1564 were the highest ethanol producing strains. The highest amount of ethanol produced was 24.85 ± 3.5 g/L achieved by Y-1564 in sweet whey (96.8% efficiency). *K. lactis* Y-1205 produced 22.39 ± 5.6 g/L ethanol in yogurt acid whey. In cream cheese acid whey, *K. lactis* strains produced significantly higher ethanol levels compared to *S. cerevisiae* and *K. marxianus* (*p* < 0.05). Outcomes from this study could provide a simple and cheap solution for small-to medium-sized dairy processing facilities to ferment lactose in whey into ethanol using lactose-consuming yeasts.

## 1. Introduction

Dairy products processing leads to the accumulation of whey by-products that result in large amounts of food waste. For every 10 L of milk used for cheese production, 9 L of liquid whey is generated (Božanić et al., 2014). The total worldwide production of whey is estimated to be approximately 200 million tons per year and increasing 1–2% each year (Buchanan et al., 2023). Crude whey contains lactose, soluble proteins, lipids, and minerals that can lead to excessive oxygen consumption and eutrophication in the environment (Zhou et al., 2019). The high organic content of this by-product poses a threat to the environment resulting in a burden on manufacturers and dairy production facilities to manage and dispose of the large amount of waste. To date, protein in crude whey can be concentrated into whey protein powders, concentrates, and isolates to decrease its environmental impacts and ease disposal (Zhou et al., 2019). Deproteinization of whey can be costly and may not be an economical solution for small-to medium-sized dairy manufacturers which make up more than 60% of USA dairy farms (Macdonald et al., 2020). Unprocessed whey has a relatively short shelf-life due to microbial spoilage. Thus, valorization of whey should consider storage, handling, and transport. Whey permeates, powders, and isolates that provide concentrated amounts of protein and lactose have been studied for the fermentation of value-added products used in the biofuel, food, biomaterials, and pharmaceutical industries (Koushki et al., 2012; Zhou et al., 2019).

Two major types of whey are generated from dairy processing: sweet whey from cheddar cheese production and acid whey from yogurt and cream cheese production. Sweet whey contains 6–9 g/L of protein, 46–52 g/L of lactose, and pH is typically between 5.6 and 7.0 (Buchanan et al., 2023). Acid whey has a lower protein content of 6–8 g/L, 44–46 g/L of lactose, and a pH of 4.3–5.6 (Buchanan et al., 2023). The composition of whey depends on processing steps and type of dairy product produced. Currently, sweet whey is processed into whey protein powders and used as a food ingredient for human consumption (Buchanan et al., 2023). Whey protein powders have increased shelf life and are easy to transport when compared to unprocessed whey. Sweet whey is processed through ultrafiltration or diafiltration to produce whey protein concentrates (WPC), whey protein powders (WPP), or whey protein isolates (WPI) (Guimarães et al., 2010). These products are used for food ingredients, biofuel, biomaterials, and pharmaceutical industries due to its economic value, ease of transport and storage, stability, and high protein content (Guimarães et al., 2010; Koushki et al., 2012; Zhou et al., 2019; Buchanan et al., 2023).

During the processing of WPC, WPP, and WPI, high volumes of lactose permeate is generated, constituting another by-product that is equivalent to the disposal of unprocessed whey (Guimarães et al., 2010). Concentration of whey can be expensive, require specialized infrastructure, and may not be economically viable for small- to medium-sized dairy manufacturers (Macdonald et al., 2020). Conversion of whey into WPC, WPP, and WPI requires more energy and water compared to liquid milk processing (Finnegan et al., 2017). While large dairy manufacturers currently employ processing techniques such as those described above to manage sweet whey, the same level of ease and efficiency is not readily achievable with acid whey. Acid whey has a chemical composition that is different from sweet whey with a lower pH, protein, and lactose content and higher calcium, phosphorous, and lactic acid which presents a challenge for membrane filtration and downstream processing (Buchanan et al., 2023). For example, large dairy manufacturers such as Chobani have implemented reverse filtration systems to recover water from acid whey (Rocha-Mendoza et al., 2021). However, an economical viable and effective downstream processing still does not exist for management of acid whey especially for small-to medium-sized facilities.

*Saccharomyces cerevisiae* is the microorganism most used for fermentation due to its ability to convert glucose into ethanol and carbon dioxide with high efficiency. Unprocessed whey contains high concentrations of the disaccharide lactose, which cannot be readily utilized by *S. cerevisiae*. Other studies have demonstrated use of enzymes or acids to hydrolyze lactose into glucose and galactose prior to fermentation with *S. cerevisiae* (Zhou et al., 2019). Other alternatives involve using engineered *S. cerevisiae* strains either through recombinant expression of *K. lactis Lac12* and *Lac4* genes, production of autolytic cells expressing the *E. coli lacZ*, or secretion of ß-galactosidase from *A. niger* (Domingues et al., 2010; Guimarães et al., 2010; Pasotti et al., 2017).

*Kluyveromyces* spp. have been known to be adapted to environments that contain lactose and demonstrate ability to convert lactose depending on the function of two genes: *Lac12* and *Lac4 (*Varela et al., 2019). Lac12 encodes a lactose permease that transports lactose into the cell and *Lac4* encodes a ß-galactosidase that further catalyzes the hydrolysis of lactose into glucose and galactose. For example, *K. marxianus* ATCC8554 can convert lactose in unprocessed whey substrate (4.9% lactose) and concentrated whey substrate (9.8% lactose) into 2.2% (w/v) and 4.6% (w/v) ethanol, respectively (Koushki et al., 2012). *K. marxianus* ETP87 isolated from yogurt was able to convert lactose from cheese acid whey (pH 3.1–4.5) into 0.6–1.2% (w/v) ethanol (Tesfaw et al., 2021). In a study by Marcus et al. (2021) inoculation of *K. marxianus* FSL B9–0008 and *K. lactis* FSL B9–0069 into deproteinized whey powder resulted in 4.52% (w/v) and 3.72% (w/v) ethanol after 20 days under anaerobic conditions (Marcus et al., 2021). *K. marxianus* DSMZ 7239 can successfully ferment lactose from liquid cheese whey in 2.5 ml batch fermenters to produce up to 20 g/L of ethanol (Christensen et al., 2011). Although many studies have demonstrated the ability of *Kluyveromyces* yeast to convert lactose to ethanol, these studies only focus on a single isolate, one type of substrate, or a combination of different yeasts or filamentous fungal species.

In this study, we selected five *Kluyveromyces* yeast inoculated into three different unprocessed whey substrates, cheddar sweet whey (CDSW), cream cheese acid whey (CCAW) and yogurt acid whey (YAW) that could be used to convert lactose into ethanol. Although *Kluyveromyces* spp. are generally thought to be lactose-consuming yeasts, we hypothesize that ethanol production will vary across selected strains and substrates. This work is the first necessary step for investigation of genetic differences and molecular mechanisms that drive ethanol production and strain specificity for optimization of whey by-products. Outcomes from this work provides a starting point for direct comparison of whole genome sequencing data, gene expression analysis, and metabolic profiling of *Kluyveromyces* spp. for lactose to ethanol conversion.

## 2. Materials and methods

### 2.1. Microorganisms

*Saccharomyces cerevisiae* BY4742 was selected as a negative control because of the absence of *Lac4* and *Lac12* genes and inability to hydrolyze lactose. *K. lactis* KB101 was a gift from Dr. Zhenglong Gu’s laboratory (Cornell University). Three *K. lactis* var. *lactis* strains (*K. lactis var. lactis* Y-62*, K. lactis var. lactis* Y-1205*, and K. lactis var. lactis* Y-1564) and one *K. marxianus* strain (*K. marxianus* Y-8281) were obtained from the NRRL Agricultural Research Service Culture Collection (ARS Culture Collection (NRRL, Peoria, IL, USA). Information on genotype, source, and background of these strains are provided in Supplementary Table 1 when available. All species were confirmed by Sanger sequencing of the internal transcribed spacer 2 (ITS2) region for yeast using primers ITS1F/ITS4R (ITS1F: 5′-TCC GTA GGT GAA CCT GCG G-3′ and ITS4: 5′-TCC TCC GCT TAT TGA TAT GC-3′). All yeast species were maintained on yeast extract peptone dextrose agar (YPDA), stored in 4°C, and re-streaked weekly.

### 2.2. Crude whey collection

Liquid whey was obtained from The Pennsylvania State University’s Berkey Creamery (State College, PA, USA). Sweet whey was collected after cheddaring and acid whey was collected after cream cheese and yogurt production. For cheddar cheese production, after the milk had been pasteurized, rennet and cultures were added to form cheese curds. During this curdling process, the curds were separated from the remaining liquid (whey) which is then drained from the tank and collected. For cream cheese production, as milk and cream were heated, lactic acid was added to lower the mixture’s pH to form curds. The curds were then heated and stabilized to form cream cheese. The liquid that remains is known as cream cheese whey and was collected as the product was cooled. For yogurt production, pasteurized milk was heated and two live cultures, *Lactobacillus bulgaricus* and *Streptococcus thermophilus* were added to thicken the milk into yogurt. The liquid that remains is known as yogurt whey and was collected directly from the fermentation tank. Liquid whey samples were processed immediately after collection by filtration through a 0.2 *μm* filter to remove starter cultures and potential spoilage microorganisms and then stored at 4°C. For the remaining of the manuscript, liquid whey used as a substrate for fermentation will be referred to as unprocessed whey.

Serial dilution and spread plating of the unprocessed and filtered whey was performed to identify potential spoilage microorganisms and starter culture organisms used during dairy processing (Supplementary Figure 1). Whey was plated onto potato dextrose agar (PDA) and Dichloran Rose Bengal Chloramphenicol agar (DRBC) to assess yeast and mold, and nutrient agar with cycloheximide was used to isolate bacteria. All plates were incubated at 30°C for 2–5 days depending on when colonies appear. All five unique colony morphologies were selected from plates. DNA extraction, PCR, and Sanger sequencing were conducted to identify the microorganisms obtained from whey (Supplementary Table 2). Fungi were sequenced using the ITS1F/ITS4R primers listed above, and bacteria were sequenced using 16S rRNA F: 5′-AGA GTT TGA TCC TGG CTC AG-3′ and 16S rRNA R: 5′-ACG GCT ACC TTG TTA CGA CTT-3′.

### 2.3. Protein content of whey substrate

Protein analysis was conducted on unprocessed and filtered CDSW, CCAW, and YAW to assess the starting protein content of whey. Filtration did not significantly alter protein concentration of the whey substrates (Supplementary Table 3). In addition, protein content was measured after 72 h of fermentation to assess whether yeast strains inoculated into different whey substrates alter protein levels. Protein analysis was conducted on a 96-well microplate assay using the Pierce™ BCA Protein Assay Kit (ThermoFisher Scientific, Rockford, IL, USA) using an Epoch 2 Microplate Reader at 562 nm (BioTek, Winnoski, VT, USA). Sample preparation and measurements were conducted based on manufacturer’s instructions. Protein was measured as BSA equivalent using a standard curve with known concentrations of BSA.

### 2.4. Micro-fermentations

**Figure 1:**
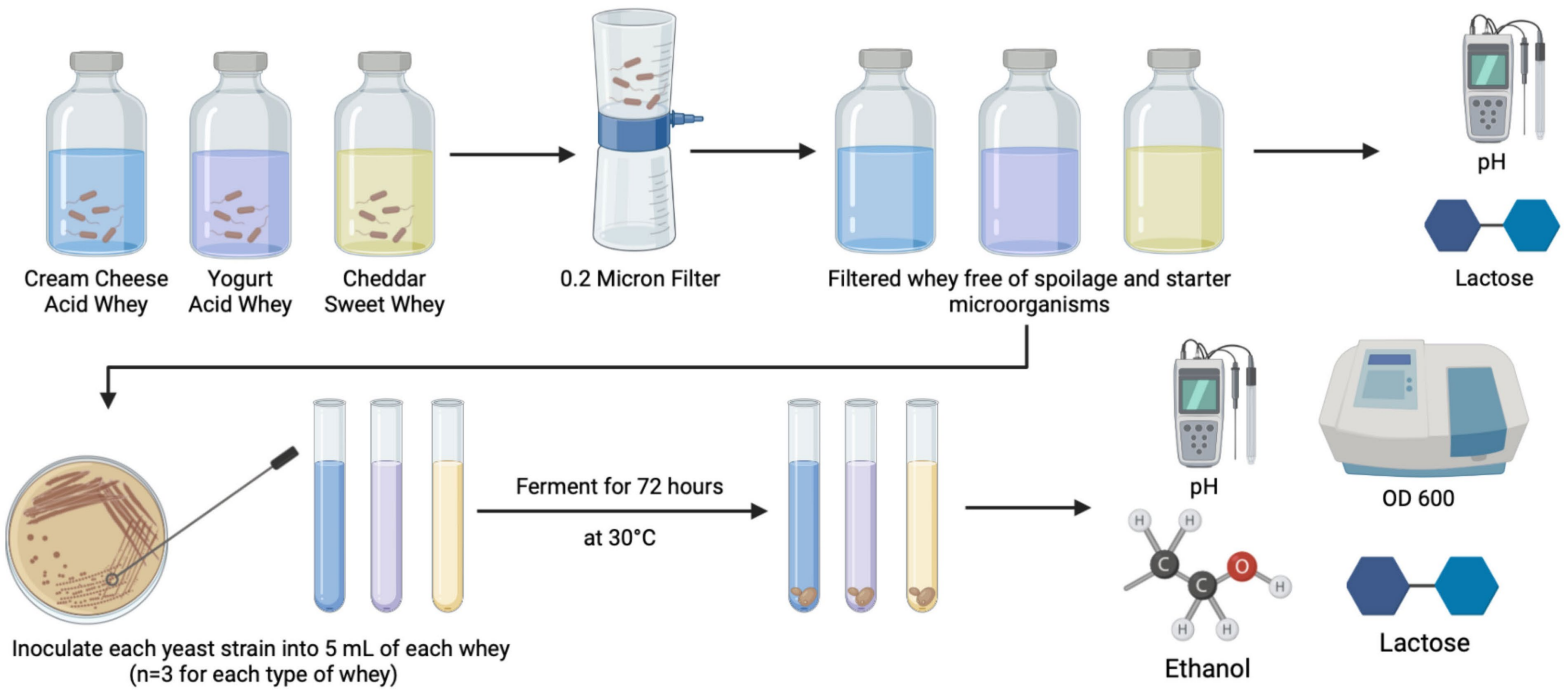
Experimental workflow for fermentation of acid and sweet whey. Whey obtained from dairy processing of cream cheese, yogurt, and cheddar cheese was filtered and used as a substrate for *Saccharomyces cerevisiae* (negative control) and five *Kluyveromyces* yeasts. Prior to the addition of yeast strains, protein levels, pH, and lactose content were measured. After 72 h, pH, lactic acid, protein levels, cell density (OD_600_), and ethanol levels were measured. Each fermentation was conducted in three independent replicates (*n* = 3).

All yeast strains were streaked onto fresh YPDA for 24 h to a colony size of approximately 1 mm. Each yeast species was individually inoculated into 5 ml of filtered CCAW, YAW, and CDSW in triplicate samples with a starting OD_600_ = 0.1 based on preliminary data generated on optimal growth curves and as previously published (Feng et al., 2021). Micro-fermentations (5 ml) were incubated at 30°C with agitation for 72 h. 30°C was chosen based on preliminary experiments and published studies (Marcus et al., 2021; Tesfaw et al., 2021). After 72 h, optical density (OD) and pH was measured using the BioTek Epoch 2 Microplate Reader (BioTek, Vinooski, VT, USA) and a FiveEasy Plus FP20 Mettler-Toledo pH meter (Mettler-Toledo, Greifensee, Switzerland), respectively. Then, micro-fermentations were filtered using a 0.2 *μm* filter for downstream analysis (lactic acid, protein, and ethanol). L-lactic acid was measured using a Unitech Scientific L-lactic acid FLEX-Reagents™ (Unitech Scientific, Hawaiian Gardens, CA, USA) and absorbance at 340 nm was read using a ChemWell^®^-T automated sampler and analyzer (Unitech Scientific). Ethanol was measured using a Megazyme K-ETOH Ethanol Assay Kit (Lesher Place, Lansing, Michigan, USA) according to the manufacturer’s instructions. A schematic of the experimental workflow is shown in Figure 1.

High-performance liquid chromatography (HPLC) was used to validate lactose concentration (g/L) of unprocessed whey prior to fermentation and candidate micro-fermentations after 72 h of CDSW and the highest performing yeast strains, *K. lactis* var. *lactis* Y-1205 and *K. lactis var. lactis* Y-1564 including *S. cerevisiae* (Supplementary Figure 2). HPLC was performed on a Vanquish Ultra High-Performance Liquid Chromatography (UHPLC) system (Thermo Fisher Scientific, Waltham, MA, USA) equipped with a RefractoMax 521 refractive index (RI) detector (Thermo Fisher Scientific, Waltham, MA, USA) at the HUCK CSL Behring Fermentation Facility at Penn State. Targeted compounds were separated and analyzed on an Aminex HPX-87H column (300 × 7.8 mm; Bio-Rad Laboratories, Hercules, CA, USA), protected by a Micro-Guard Cation H guard column (30 × 4.6 mm; Bio-Rad Laboratories, Hercules, CA, USA) and kept at 60°C. The analytical conditions used were as follows: 10 μl of injection volume, flow 0.5 ml/min, eluent 5 mM H_2_SO_4._ Temperatures set for autosampler, and RI detector were 4 and 35°C, respectively. A standard curve was prepared using standards to determine the relationship between concentration and the peak area of a particular compound eluted. The peak on each chromatogram corresponding to each compound was identified by comparing the retention time with that of standards. All standards and samples were injected in technical triplicate.

Theoretically, 1 pound of lactose will yield 0.538 pounds of ethanol. The lactose in whey (4.5–5%) would therefore yield around 2.5% ethanol when assuming 100% efficiency (Koushki et al., 2012). Fermentation efficiency was calculated by ethanol production (%) per theoretically maximum ethanol production (2.5%) multiplied by 100.

To assess growth rates, growth curves were generated using a microplate assay. Each yeast strain was inoculated into 200 μl of different whey substrates with a starting OD_600_ of 0.1 in a 96-well plate (n = 3 wells per substrate for each strain; Supplementary Figure 3). The 96-well plate was incubated at 30°C with agitation for 72 h and OD was measured every 90 min using the BioTek Epoch 2 Microplate Reader (BioTek, Vinooski, VT, USA). Growth rate, V_max_ (log OD versus time) which is the slope of the portion of the growth curve was used to compare fermentation parameters between strains inoculated into different whey substrates over 72 h.

### 2.5. Statistical analysis

A one-way ANOVA followed by a Tukey’s HSD or Dunnett’s multiple comparisons test was performed to determine statistical significance between experimental replicates (n = 3) for strains and different whey substrates (*p* < 0.05). Statistical analyses were performed using the Minitab software (Minitab LLC, 2021) or GraphPad Prism version 9.5.1 for Mac, GraphPad Software, San Diego, California USA, www.graphpad.com.

## 3. Results

### 3.1. Evaluation of pH and lactic acid concentration in whey substrates after 72 h

The pH of sweet and acid whey was measured before and after 72 h to assess pH changes in different whey substrates inoculated with *Kluyveromyces* spp. and *S. cerevisiae* (Table 1). In CDSW, all strains decreased pH significantly from the starting pH of 6.41 (*p* < 0.05), except *K. marxianus*. The magnitude of pH decrease for *K. lactis, K. lactis* Y-1205, and *K. lactis* Y-1564 was the highest; decreasing the starting pH of CDSW from 6.41 to 5.22, 5.13, and 5.28, respectively. Similarly, in CCAW, *K. lactis, K. lactis* Y-1205, and *K. lactis* Y-1564 decreased pH significantly from 4.83 to 4.50, 4.55, and 4.54, respectively, (*p* < 0.05). *S. cerevisiae* did not change the pH of CCAW as pH remained the same or close to the starting pH. We observed an increase in pH of CCAW after 72 h in CCAW substrate inoculated with *K. marxianus* (*p* < 0.05). In YAW, all strains significantly decreased the starting pH of 4.79 to 4.48–4.58 (*p* < 0.05). After 72 h, *Kluyveromyces* spp. and *S. cerevisiae* inoculated into YAW resulted in significant decreases of pH ranging between 0.2–0.3 when compared to the starting pH.

**Table 1:** pH and change in pH after 72 h of fermentation with different whey substrates using *Kluveromyces* spp. compared to *S. cerevisiae*.

	CDSW starting pH = 6.41		CCAW starting pH = 4.83		YAW starting pH = 4.79	
	pH	Δ pH	pH	Δ pH	pH	Δ pH
*S. cerevisiae*	6.260 ± 0.010	−0.150*	4.843 ± 0.054	0.013	4.483 ± 0.023	−0.307*
*K. lactis*	5.220 ± 0.010	−1.190*	4.507 ± 0.018	−0.323*	4.480 ± 0.000	−0.310*
*K. lactis* Y-62	5.920 ± 0.021	−0.490*	4.787 ± 0.027	−0.043	4.583 ± 0.013	−0.207*
*K. lactis* Y-1205	5.130 ± 0.023	−1.280*	4.550 ± 0.006	−0.280*	4.520 ± 0.012	−0.270*
*K. lactis* Y-1564	5.280 ± 0.032	−1.130*	4.540 ± 0.012	−0.290*	4.560 ± 0.017	−0.230*
*K. marxianus*	6.273 ± 0.066	−0.137	5.200 ± 0.120	0.370*	4.527 ± 0.009	−0.263*

pH values are means ± standard deviation of three independent replicates. Change in pH is calculated by the difference between starting and final pH; negative values represent a drop in pH, positive values represent increase in pH. Values with asterisks (*) represent a significant change compared to the starting pH of whey (Tukey HSD test, *p* < 0.05). CDSW; cheddar cheese sweet whey, CCAW; cheddar cheese acid whey, YAW; yogurt acid whey.

**Figure 2:**
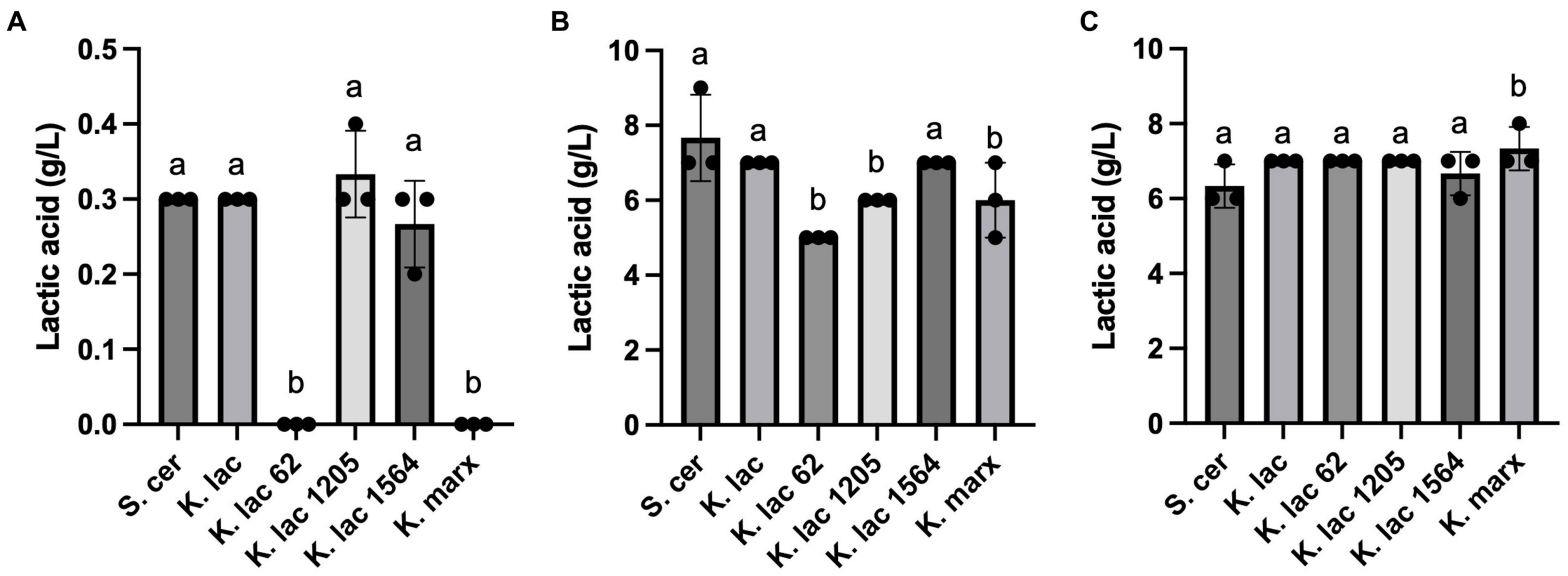
Lactic acid levels measured after 72 h in **(A)** cheddar cheese sweet whey, **(B)** cream cheese acid whey, and **(C)** yogurt acid whey inoculated with five *Kluyveromyces* yeasts compared to *S. cerevisiae*. Bar graphs represent means ± standard deviations of three independent replicates. Different letters indicate that difference is significant compared to the control, *S. cerevisiae* (one-way ANOVA followed by a Dunnett’s multiple comparison test, *p* < 0.05). Note that the scale for y-axis of panel **(A)** was enlarged and is different than the y-axis scale of panel **(B,C)**.

Lactic acid concentration in whey substrates were measured after 72 h (Figure 2). Compared to the negative control, *S. cerevisiae*, no detectable levels of lactic acid were observed in CDSW inoculated with *K. lactis* Y-62 and *K. marxianus* (Figure 2A). No significant differences were detected between lactic acid concentration in CDSW inoculated with *K. lactis*, *K. lactis Y-1205, and K. lactis* Y-1564 compared to the control. In CCAW, we observed significantly lower levels of lactic acid in CCAW inoculated with *K. lactis* Y62, *K. lactis* Y-1205, and *K. marxianus* when compared to the control (*p* < 0.05; Figure 2B). However, lactic acid levels of CCAW inoculated with *K. lactis and K. lactis Y-1564* were not significantly different when compared to the control (Figure 2C). In YAW, no significant difference was detected in lactic acid levels between *K. lactis* strains when compared to the control except for *K. marxianus* where a 1 g/L increase was observed.

### 3.2. Protein analysis of different whey substrates before and after 72 h fermentation

**Figure 3:**
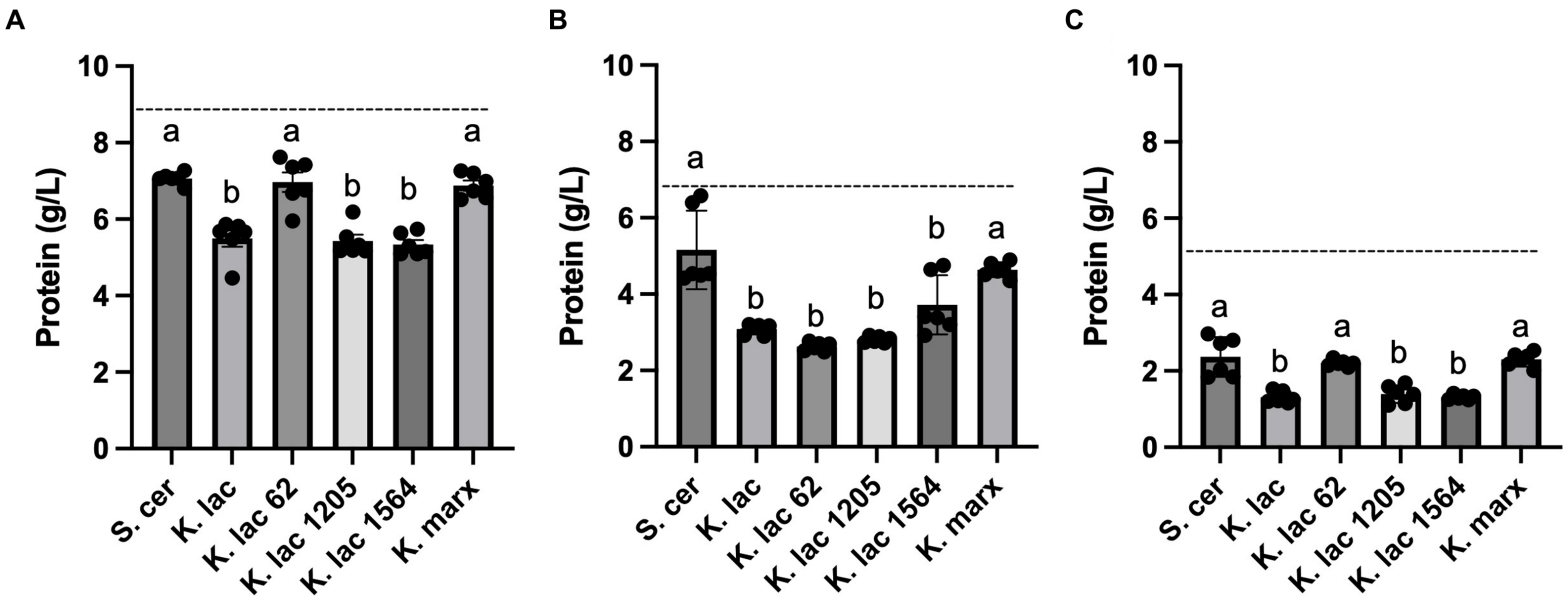
Protein concentration measured after 72 h in **(A)** cheddar cheese sweet whey, **(B)** cream cheese acid whey, and **(C)** yogurt acid whey inoculated with five *Kluyveromyces* yeasts compared to *S. cerevisiae*. Bar graphs represent means ± standard deviations of three independent replicates. Different letters indicate that difference is significant compared to the control, *S. cerevisiae* (one-way ANOVA followed by a Dunnett’s multiple comparison test, *p* < 0.05). The dotted line across each graph represents the starting protein concentration for each whey substrate: cheddar cheese sweet whey, 8.67 g/L; cream cheese acid whey, 6.36 g/L; yogurt acid whey, 5.26 g/L.

The starting protein concentration of CDSW (8.67 g/L) is higher when compared to that of CCAW (6.36 g/L) and YAW (5.26 g/L). We observed that *K. lactis*, *K. lactis* Y-1205, and *K. lactis* Y-1564 significantly decrease protein levels in CDSW and YAW when compared to the control, *S. cerevisiae* (Figures 3A,C). In CCAW, all *K. lactis* strains significantly decrease protein levels in the whey substrate compared to the control (Figure 3B). In all whey substrates tested, no significant differences were detected in protein levels between *S. cerevisiae* and *K. marxianus* after 72 h. Although all yeast strains decrease protein levels after 72 h of fermentation, the magnitude of decrease is dependent on the whey substrate and yeast strains used.

### 3.3. Monitoring of cell density and growth rates as an indicator of fermentation efficiency

**Figure 4:**
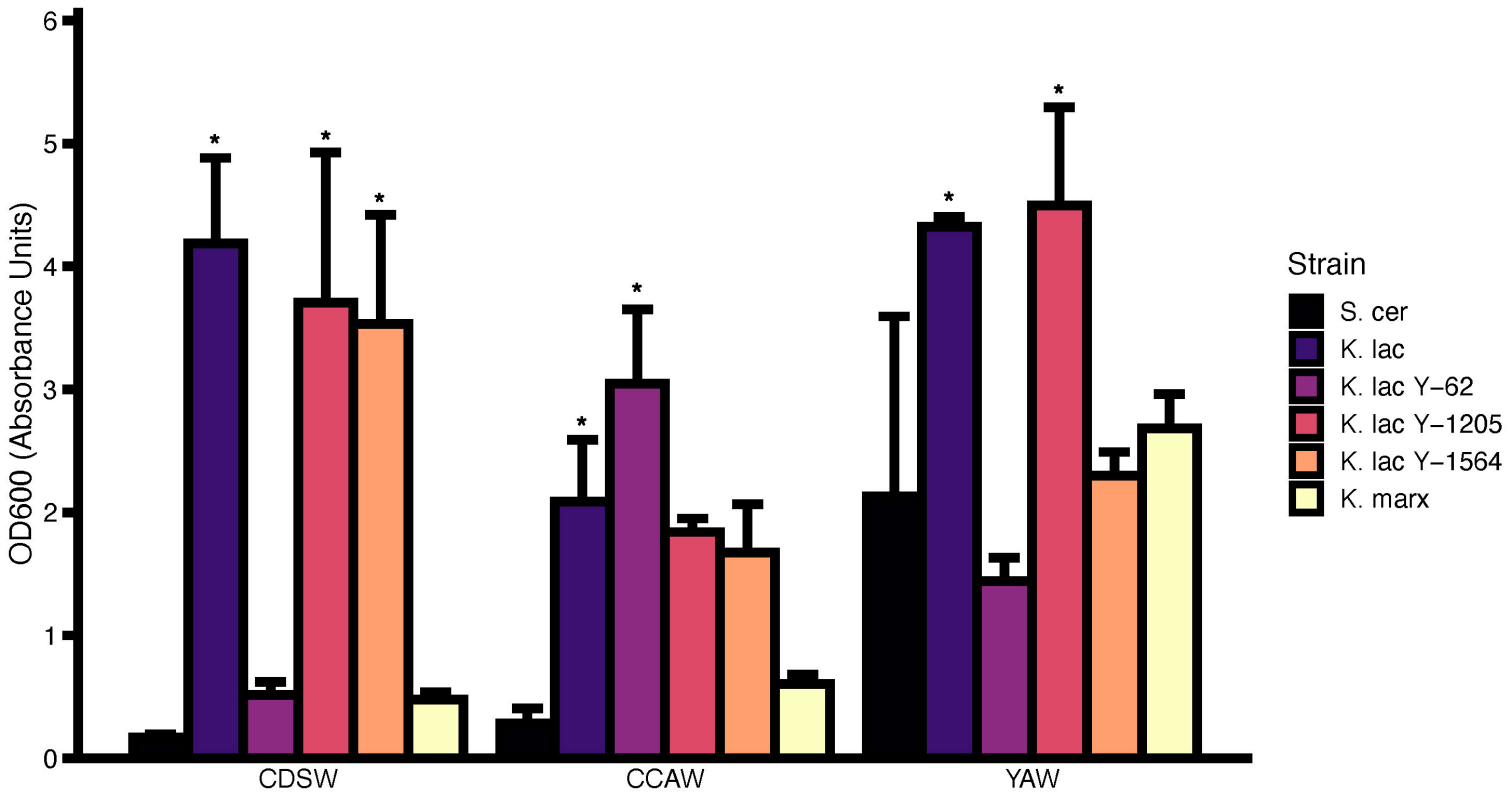
Cell density (OD_600_) measurements of cheddar cheese sweet whey (CDSW), cream cheese acid whey (CCAW), and yogurt acid whey (YAW) inoculated with five *Kluyveromyces* yeasts compared to *S. cerevisiae* after 72 h. Initial fermentations were standardized with a starting OD_600_ = 0.1 which is approximately equivalent to 10^6^–10^7^ CFU/ml. Bar graphs represent means ± standard error of the mean. Yeast strains with asterisks (*) are significantly different from the negative control within the specified substrate (*S. cerevisiae*; Tukey’s test, *p* < 0.05).

OD_600_ was measured after 72 h with the initial OD_600_ of the six candidate yeast strains standardized to 0.1 (Figure 4). We reasoned that an increase in cell density after fermentation would be a good indicator for microbial growth. In CDSW, *K. lactis, K. lactis* Y-1205, and *K. lactis* Y-1564 had significantly higher OD_600_ values from OD_600_ = 0.1 to OD_600_ = 3.53–4.19 (*p* < 0.05) compared to the control, *S. cerevisiae*. The OD_600_ of *K. lactis* Y-62 and *K. marxianus* were not significantly different than *S. cerevisiae* (*p* > 0.05). We observed significantly higher OD_600_ after 72 h of CCAW inoculated with *K. lactis* and *K. lactis* Y-62 (*p* < 0.05). In YAW, *K. lactis* and *K. lactis* Y-1205 had significantly higher OD_600_ compared to other yeast strains reaching 4.32 and 4.50, respectively (*p* < 0.05)*. S. cerevisiae* demonstrated a 10-fold increase in OD_600_ (OD = 2.13) in YAW compared to CCAW and CDSW.

In addition to OD_600_, growth rate (log OD versus time) was used to compare fermentation parameters between strains inoculated into different whey substrates over 72 h using a microplate assay (Table 2). Compared to *S. cerevisiae, K. lactis* Y-1205 and *K. lactis* Y-1564 demonstrate significantly higher growth rates in CDSW (*p* < 0.05). In CCAW, only *K. lactis* Y-1205 showed significantly higher growth rates compared to the control. Strains that grew the best in YAW substrate were *K. lactis* Y-1205, *K. lactis* Y-1564, and *K. marxianus*. In sum, higher OD_600_ and growth rates were observed in CDSW and YAW compared to CCAW.

**Table 2:** Growth rates of *Kluyveromyces* spp. compared to *S. cerevisiae* in different whey substrates measured every 90 min over 72 h using a 96-well microplate assay.

Strain	Growth rate* (h^−1^)		
	CDSW	CCAW	YAW
*S. cerevisiae*	0.404 ± 0.3^a^	0.165 ± 0.02^a^	0.207 ± 0.06^a^
*K. lactis*	1.091 ± 0.6^a^	0.824 ± 0.3^a^	1.095 ± 0.1^a^
*K. lactis* Y-62	0.609 ± 0.3^a^	0.208 ± 0.1^a^	1.065 ± 0.2^a^
*K. lactis* Y-1205	1.946 ± 0.1^b^	1.166 ± 0.8^b^	1.598 ± 0.2^b^
*K. lactis* Y-1564	1.892 ± 0.2^b^	0.190 ± 0.02^a^	2.102 ± 0.7^c^
*K. marxianus*	0.930 ± 0.5^a^	0.251 ± 0.01^a^	1.874 ± 0.3^c^

Values are means ± standard deviations of three independent replicates. Shared superscript letters in the same column indicate no significant difference; different superscript letters in the same column indicate that difference is significant compared to the control, *S. cerevisiae* (Dunnett’s test, *p* < 0.05). CDSW; cheddar cheese sweet whey, CCAW; cheddar cheese acid whey, YAW; yogurt acid whey.

* Growth rate was obtained as the slope of the linear portion of the growth curve expressed as log OD over time.

### 3.4. Type of whey combined with *Kluyveromyes* spp. is important for optimization of ethanol production

**Figure 5:**
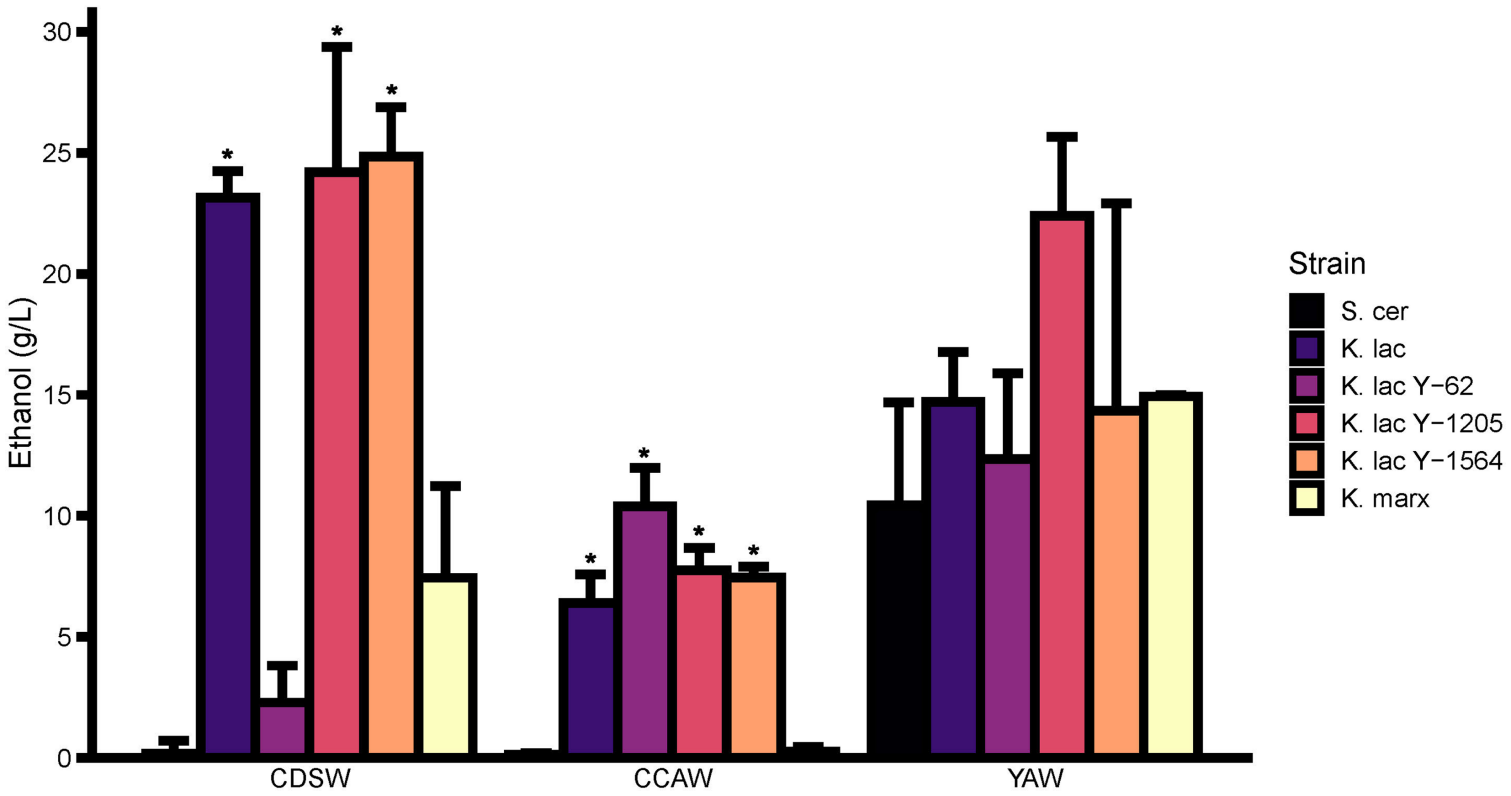
Comparison of ethanol concentration between cheddar cheese sweet whey (CDSW), cream cheese acid whey (CCAW), and yogurt acid whey (YAW) inoculated with five *Kluyveromyces* yeasts compared to *S. cerevisiae* after 72 h. Ethanol levels were measured using a Megazyme Ethanol Microplate Assay kit using an internal ethanol standard curve. Bar graphs represent means ± standard error of the mean. Yeast strains with asterisks (*) are significantly different from the negative control within the specified substrate (*S. cerevisiae*; Tukey’s test, *p* < 0.05).

Ethanol was measured for six candidate yeast strains in each whey substrate after 72 h (Figure 5). In CDSW, *K. lactis, K. lactis* Y-1205, and *K. lactis* Y-1564 produced significantly higher amounts of ethanol than the negative control at 23.1–24.8 g/L of ethanol (p < 0.05). *K. lactis* produced 23.15 ± 1.9 g/L ethanol while *K. lactis* Y-1205 and *K. lactis* Y-1564 produced 24.20 ± 9.0 g/L and 24.85 ± 3.5 g/L ethanol in CDSW, respectively. *K. lactis* Y-62 and *K. marxianus* did not produce significantly different ethanol levels when compared to *S. cerevisiae* (*p* > 0.05). In CCAW, *K. lactis, K. lactis* Y-62, *K. lactis* Y-1205, and *K. lactis* Y-1564 produced significantly higher ethanol levels when compared to *S. cerevisiae* ranging between 6.4 and 10.4 g/L (*p* < 0.05). In this acid whey substrate, we did not detect any differences in ethanol levels between *K. marxianus* and *S. cerevisiae* (*p* > 0.05). In YAW, none of the candidate yeast strains had significantly different ethanol concentrations from each other when compared to *S. cerevisiae* (*p* > 0.05). The highest ethanol-producing strain in YAW was *K. lactis* Y-1205 which produced up to 22.4 ± 5.7 g/L of ethanol.

To access fermentation efficiency, we selected CDSW and the two highest producing ethanol strains *K. lactis* Y-1205 and *K. lactis* Y-1564 as well as the negative control *S. cerevisiae* for calculation of lactose utilization, ethanol production, and fermentation efficiency. Fermentation efficiency was then calculated for the three strains (Table 3). *S. cerevisiae* showed the lowest fermentation efficiency at 0.74%. *K. lactis* Y-1205 and *K. lactis* Y-1564 demonstrated high fermentation efficiencies at 96.8 and 99.4% in CDSW, respectively.

**Table 3:** Fermentation efficiency of *S. cerevisiae, K. lactis Y-1205*, and *K. lactis Y-1564* after 72 h fermentation in cheddar cheese sweet whey.

Strain	Lactose (% w/v)	Lactose utilized by yeast (%)	Ethanol production (% w/v)	Fermentation efficiency (%)
*S. cerevisiae*	5.4	2.55	0.02	0.74
*K. lactis* Y-1205	5.4	97.32	2.42	96.79
*K. lactis* Y-1564	5.4	99.78	2.48	99.39

## 4. Discussion

Sustainability, energy security, and economic drivers have increased dairy manufacturer’s interests in assessing alternative technologies that enable valorization of dairy by-products such as whey. The concept of producing bioethanol from whey is not novel and has previously been conducted (Guimarães et al., 2010; Koushki et al., 2012; Marcus et al., 2021; Tesfaw et al., 2021). Whey can be concentrated into WPP, and then the remaining lactose permeate is fermented to produce bioethanol. This process includes ultrafiltration of sweet whey, concentration of the lactose permeates through reverse osmosis, and then fermentation of lactose. Once fermentation is complete, the resulting liquid undergoes distillation to recover ethanol. The remaining liquid and biomass are then sent to a treatment facility as they are still considered environmental pollutants or further processed into food ingredients, animal feed, or other products. Despite its effectiveness, the adoption of this method by dairy manufacturers has been limited due to high processing costs and its restriction to sweet whey only. In this study, we propose conversion of lactose to ethanol for downstream processing and management of sweet and acid whey that is simple and cheap to deploy especially for small-to medium-sized facilities.

Previous studies have demonstrated the ability of microorganisms including *Kluyveromyces* yeast to convert lactose to ethanol (Guimarães et al., 2010; Pasotti et al., 2017; Marcus et al., 2021; Tesfaw et al., 2021). However, these studies either focus on a single isolate, one type of substrate, or a combination of different yeasts or filamentous fungal species. Direct comparison of previous application of *Kluyveromyces* spp. in whey fermentation is challenging due to strain specificity, lack of whole genome sequencing data, and molecular mechanisms that could help explain differences observed in fermentation efficiency (lactose to ethanol conversion). Thus, in our work we systematically selected only *Kluyveromyces* spp. and tested these strains against three different whey substrates to form the basis for detailed downstream mechanistic understanding on the association between genotype to phenotype.

We demonstrate that *K. lactis* strains were more effective than *K. marxianus* at converting lactose to ethanol in all whey substrates. In CDSW, the yeast strains *K. lactis* Y-1205 and *K. lactis* Y-1564 produced the highest concentrations of ethanol with high fermentation efficiencies: *K. lactis* Y-1205 produced 29.6 g/L of ethanol at 96.8% efficiency, and *K. lactis* Y-1564 produced 28.9 g/L of ethanol at 99.4% efficiency. In contrast to our findings, previous studies have found that *K. marxianus* can produce high concentrations of ethanol in whey substrates (Božanić et al., 2014; Marcus et al., 2021). *K. marxianus* has been previously reported to produce ethanol levels as high as 26 g/L in a 6.5% lactose solution using deproteinized whey (Guimarães et al., 2010). Another study demonstrated that *K. marxianus* can produce 2.2% ethanol (w/v) from a 4.9% lactose whey permeate (Koushki et al., 2012). One possible explanation could be the three *K. marxianus* haplotype that exists; A, B, and C. Only haplotype B has been shown to hydrolyze lactose (Varela et al., 2019). All three haplotypes contain *Lac12* and *Lac4*, but haplotypes A and C encode a nonfunctional *Lac12*. We speculate that the NRRL strain (*K. marxianus Y-*8281) used in this study is a haplotype with a non-functional *Lac12* gene. A combination of whole genome sequencing and RNA gene expression analysis can help explain differences in lactose metabolism across *Kluveromyces* spp. in future studies.

Sweet whey (CDSW) appears to be a preferred substrate for the highest production of ethanol compared to acid whey, YAW and CCAW. One explanation could be the differences in chemical composition of whey substrates. For example, protein content of CDSW is typically higher (8.67 g/L; 0.8% w/v) compared to acid whey (5.26–6.36 g/L; 0.5–0.6% w/v). These ranges are in line with the levels obtained by other studies (Tesfaw, 2023). After 72 h, protein content decreased significantly in CDSW inoculated with *K. lactis, K. lactis* Y-1205, and *K. lactis* Y-1564. These strains also produce the highest amount of ethanol in CDSW. It is possible that the decrease in pH enhances proteolysis of whey proteins and this favored lactose to ethanol conversion. Proteolytic activity has been previously reported in *K. lactis* NRRL 1118 and Zhang et al. showed that *K. marxianus* can convert caseins and bovine serum proteins in milk into bioactive peptides (Flores et al., 1999; Zhang et al., 2017). Although the effect of protein hydrolysis on lactose to ethanol conversion is beyond the scope of this study, it would be an interesting next step to profile free amino acids and volatile organic compounds. Increase in free amino acids, bioactive peptides, and volatile organic compounds could be a route for development of a functional fermented beverage.

YAW was the only substrate where all yeast species were able to produce ethanol, including *S. cerevisiae*. *S. cerevisiae* and *K. marxianus* were able to grow in YAW (higher OD_600_ compared to CDSW and CCAW) and subsequently produced ethanol in YAW. One explanation can be explained by the activity of starter culture microorganisms (*Lactobacillus* and *Streptococcus* spp.) and collection of whey directly from the yogurt fermentation tank. During yogurt production, starter cultures hydrolyze lactose into glucose and galactose. This was evidenced on HPLC chromatographs: glucose and galactose were detected in YAW prior to fermentation but not in CDSW (Supplementary Figures 2A, 4). Therefore, *S. cerevisiae* could convert the glucose and galactose present in YAW to ethanol. Although not significant when compared to *S. cerevisiae*, *K. lactis* Y-1205 produced the highest amounts of ethanol in YAW. This strain may be a good candidate for use in lactose to ethanol conversation of YAW since the downstream processing of acid whey is a bigger challenge to dairy manufacturers (Buchanan et al., 2023).

Most yeast strains used in this study decreased the pH of all three whey substrates after 72 h of fermentation. This could be partially explained by lactic acid levels measured after 72 h. For example, the small magnitude of decrease (~0.3 g/L) between *K. lactis* Y-1205 and Y-1564 when compared to Y-62 and *K. marxianus* does not fully explain the change in pH (decrease of 1.1–1.3). One exception to our observation is the increase in pH of CCAW with *K. marxianus*. Under anaerobic conditions, *K. marxianus* increased acetic acid levels in whey permeate from 0.66 g/L at day 0 to 1.17 g/L at day 20 and 1.39 g/L at day 34 (Marcus et al., 2021). However, in the same study, *K. marxianus* decrease lactic acid levels in whey from 0.79 g/L at day 0 and 20 to 0.47 g/L at day 34. The authors also determined that levels of other organic acids such as tartaric acid and malic acid were low to non-detectable. One possible explanation to the increase in pH is protein hydrolysis by *K. marxianus*. One limitation in our study is the volume and size of 5 ml micro-fermentation. While micro-fermentations allowed rapid screening of multiple strains and substrates simultaneously, our next step is to increase the volumes to 500 ml and 5 L fermentations to validate observations from this study as it relates to ethanol production. Another limitation is the need to filter sterilize whey substrates to control for the effect of starter and potential spoilage microorganisms on lactose to ethanol conversion by *Kluyveromyces* yeasts. As a follow up study, we are using HTST pasteurization of whey to reduce microbial load prior to fermentation. This approach would be more economical and practical for the dairy industry compared to microfiltration.

## 5. Conclusion

In conclusion, we demonstrate that differences exist in ethanol production across different sweet and acid whey substrates inoculated with *Kluyveromyces* yeast. In sweet whey, *K. lactis, K. lactis* Y-1205 and *K. lactis* Y-1564 were the highest ethanol producing strains. The highest amount of ethanol produced was 24.85 ± 3.5 g/L achieved by Y-1564 in CDSW. One simple and cheap strategy for small-to medium-sized dairy manufacturers could be to inoculate whey using *K. lactis* Y-1564 to convert lactose into bioethanol. With the absence of a technoeconomic analysis and life cycle assessment, it is uncertain whether bioethanol should be further processed for use as an energy source or used directly as a fermented beverage. However, outcomes from this work provides a starting point for direct comparison of whole genome sequencing data, gene expression analysis, and metabolic profiling of *Kluyveromyces* spp. for lactose to ethanol conversion of whey by-products from dairy processing.

## Data availability statement

The original contributions presented in the study are included in the article/Supplementary material, further inquiries can be directed to the corresponding author.

## Author contributions

AMO, XD, and JW conceived and designed the study. AMO performed all experiments and statistical analysis and wrote the manuscript with input from JW. AEB performed microbial characterization of whey. JW supervised the project. All authors discussed the results and contributed to manuscript revision, read, and approved the submitted version.

## Funding

AMO was supported by an undergraduate research award from the Penn State College of Agricultural Sciences and the Department of Food Science. AEB was supported by the USDA-funded Research and Extension Experiences for Undergraduates (REEU) project “Bugs in my food: research and professional development in food safety for undergraduates from non-land grant institutions” (USDA-NIFA grant 2021-67037-34628). This work is supported by the USDA National Institute of Food and Agriculture and Hatch Appropriations under Project #PEN04699 and Accession #1019351 to JW and a College of Agricultural Sciences SNIP grant to JW.

## Conflict of interest

The authors declare that the research was conducted in the absence of any commercial or financial relationships that could be construed as a potential conflict of interest.

## Publisher’s note

All claims expressed in this article are solely those of the authors and do not necessarily represent those of their affiliated organizations, or those of the publisher, the editors and the reviewers. Any product that may be evaluated in this article, or claim that may be made by its manufacturer, is not guaranteed or endorsed by the publisher.
