# Supplementary material for: Evaluation of Kluyveromyces spp. for conversion of lactose in different types of whey from dairy processing waste into ethanol
Source: Front Microbiol. 2023 Aug 8;14:1208284. doi: 10.3389/fmicb.2023.1208284 (PMC10442841; doi:10.3389/fmicb.2023.1208284)
Supplement: Supplementary file 1 [file Data_Sheet_1.pdf]

# *Supplementary Material*

# Evaluation of *Kluyveromyces* spp. for conversion of lactose in different types of whey from dairy processing waste into ethanol

**Ashley Ohstrom[1], Autumn Buck[1], Xue Du[1], Josephine Wee[1*]**

*** Correspondence:** Corresponding Author: jmw970@psu.edu

## 1 Supplementary Data

**Supplementary Table 1** Yeast strains, designation, isolation information (genotype), and source

| Strain | Strain designation | Isolation information (genotype) | Source |
| --- | --- | --- | --- |
| *Saccharomyces cerevisiae* | BY4742 | Derived from S288C (MATα his3Δ1 leu2Δ0 lys2Δ0 ura3Δ0) | Unknown |
| *Kluyveromyces lactis var. lactis* | KB101 | Dairy environment (MATα ade trpl ura3gal80-1) | Gu, Cornell University, Ithaca, New York |
| *Kluyveromyces lactis var. lactis* | NRRL: Y-62 | DFD, soft cheese, Tanner collection, Italy | Clark, University of Illinois, Urbana, Illinois |
| *Kluyveromyces lactis var. lactis* | NRRL: Y-1205 | Unknown, Allen, Soriano | Laffer, University of Illinois, Urbana, Illinois |
| *Kluyveromyces lactis var. lactis* | NRRL: Y-1564 | DFD, soft cheese, Italy | Kreger-van Rij, CBS, Baarn, The Netherlands |
| *Kluyveromyces marxianus* | NRRL: Y-8281 | Unknown | D. Yarrow, CBS, Delft, The Netherlands |

**Supplementary Table 2** Starter and spoilage microorganisms identified from unfiltered whey. All five unique colony morphologies from serial dilution and plating were identified by DNA extraction, PCR, and Sanger sequencing.

| Starter Microorganisms | Spoilage Microorganisms |
|---|---|
| *Galactomyces geotrichum* | *Rhodotorula mucilaginosa* |
| *Kluyveromyces lactis* | *Torulaspora delbrueckii* |

**Supplementary Table 3** Protein concentration for acid whey, filtered acid whey, sweet whey, and filtered sweet whey.

| Acid whey | Filtered acid whey | Sweet whey | Filtered sweet whey |
|---|---|---|---|
| 5.84 ± 0.51[a] | 5.99 ± 0.42[a] | 9.46 ± 0.40[b] | 8.68 ± 0.70[b] |

Protein concentration (g/L) are presented as means ± standard deviation of three independent replicates (n=3). Samples that do not share the same letter are significantly different.

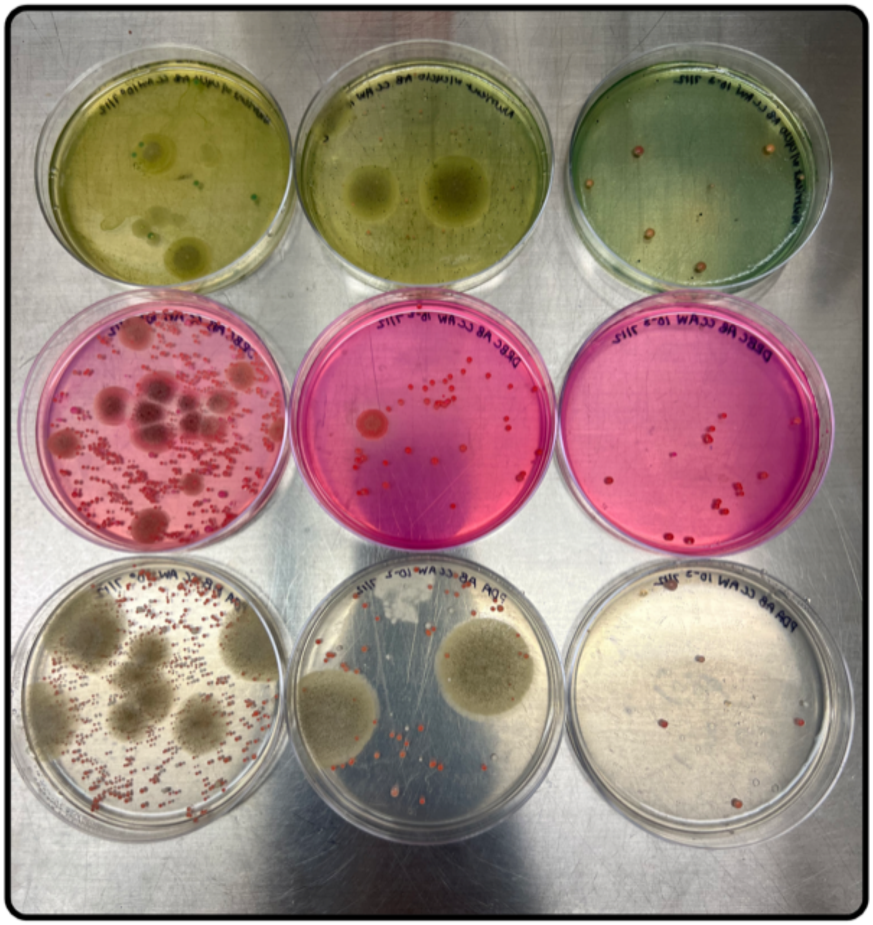

**Supplementary Figure 1** Microorganisms from unfiltered whey on potato dextrose agar (PDA; bottom row), Dichloran Rose Bengal Chloramphenicol agar (DRBC; middle row), and nutrient agar with cycloheximide (top row).

**A**

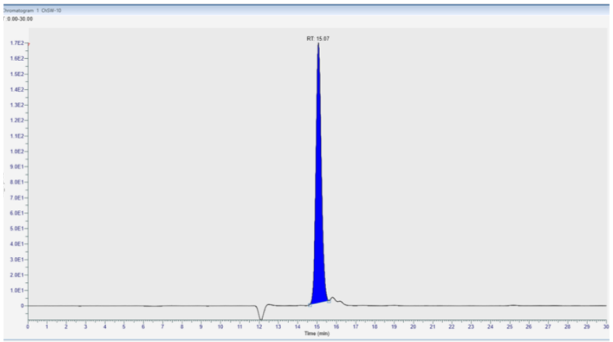

**B**

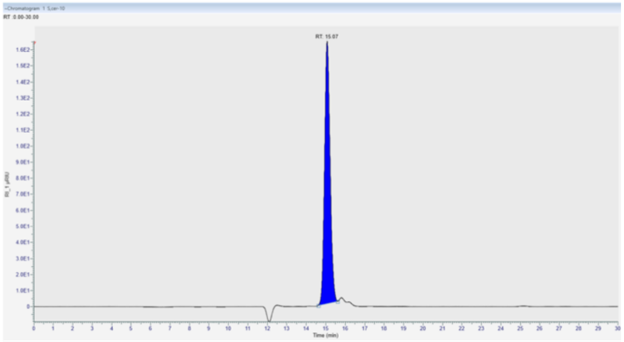

**C**

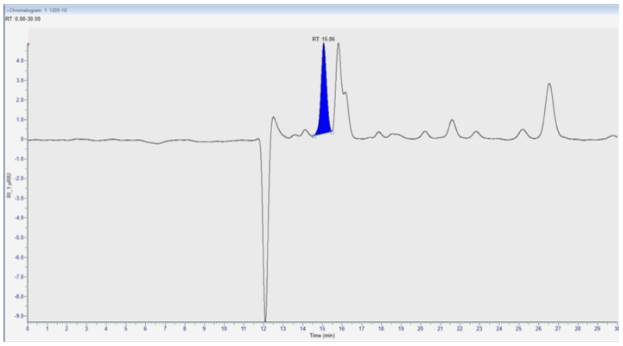

**D**

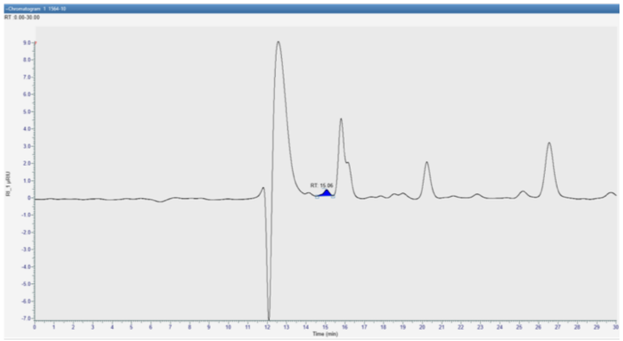

**Supplementary Figure 2** HPLC chromatograms of (A) uninoculated cheddar sweet whey (CDSW), (B) cheddar sweet whey after 72 h with *S. cerevisiae*, (C) CDSW after 72 h inoculated with *K. lactis* Y-1205, and (D) CDSW after 72 h inoculated with *K. lactis* Y-1564. Lactose is represented by the peaks with blue area under the curve (retention time, RT of 15.06-15.07).

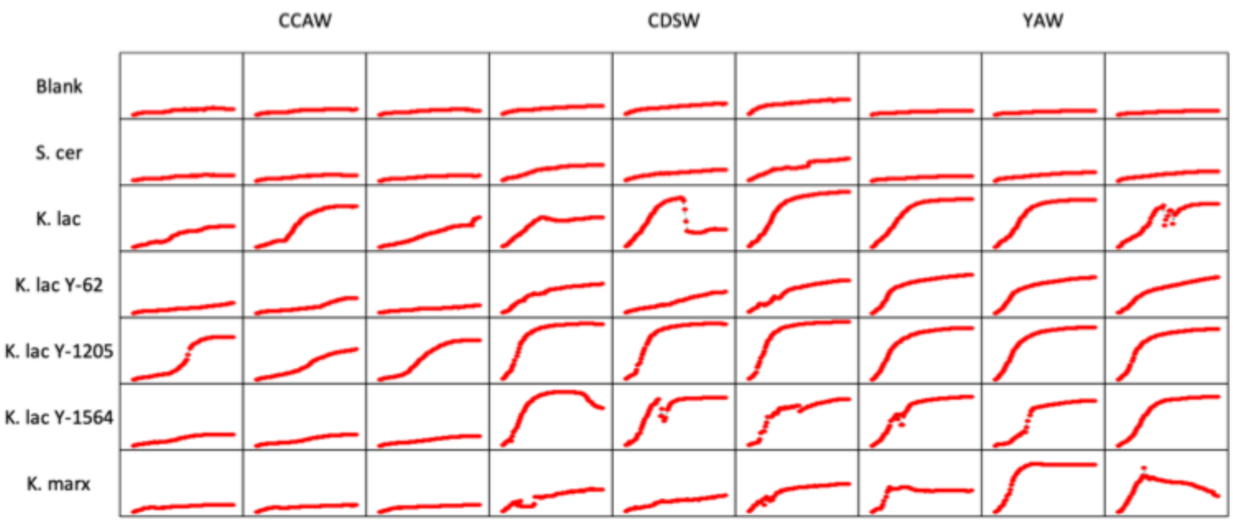


**Supplementary Figure 3** Representative growth curves (n=3) of five *Kluyveromyces* yeasts and *S. cerevisiae* in cream cheese acid whey (CCAW), cheddar sweet whey (CDSW) and yogurt acid whey (YAW) every 90 mins for 72 hours using the Biotek Epoch2 Microplate Reader.

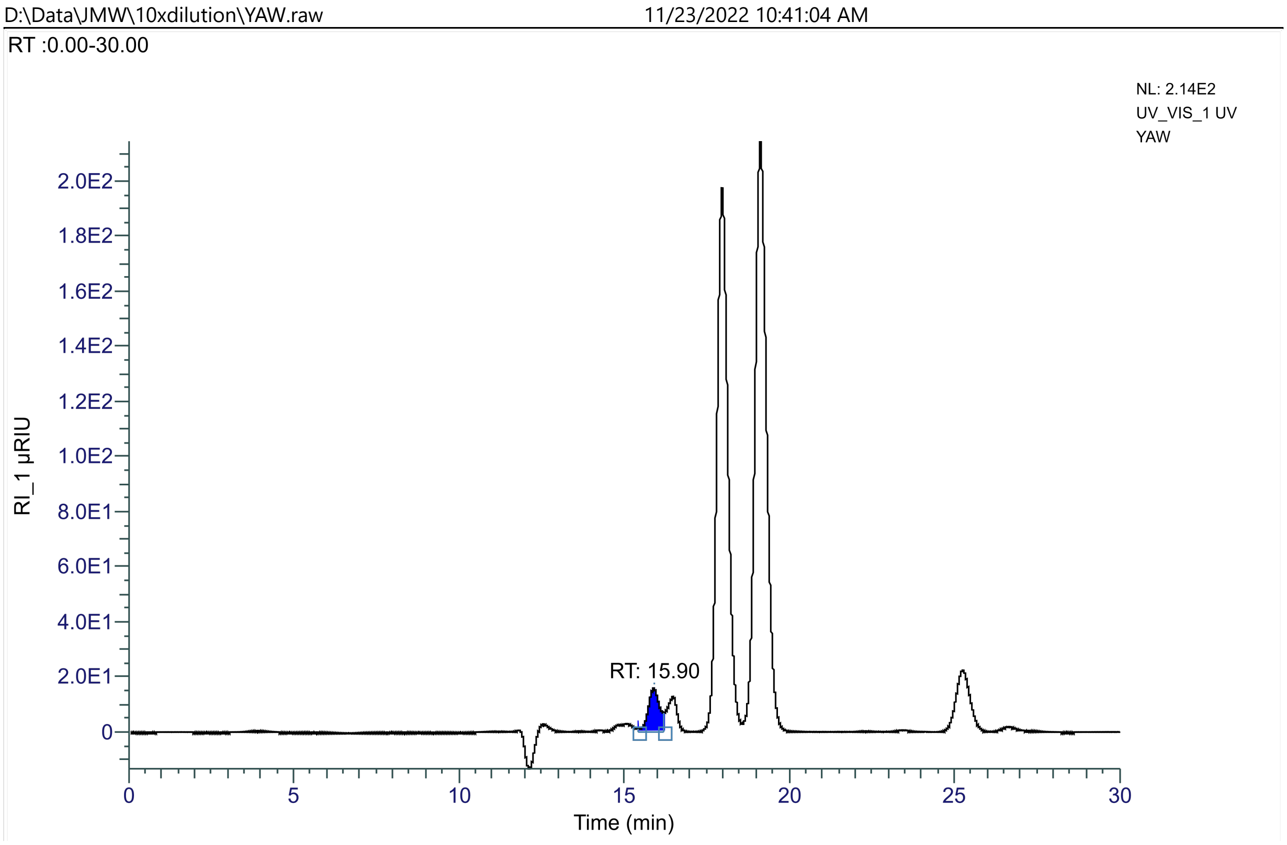


**Supplementary Figure 4** HPLC lactose chromatogram of uninoculated yogurt acid whey. The highlighted peak represents lactose (RT: 15.90), and the other two peaks correspond to glucose and galactose.
